# Tumor-intrinsic immune-genetic dynamics identify RNASE1 as an immune-evasion-associated biomarker and predictor of checkpoint blockade response in gastric adenocarcinoma

**DOI:** 10.3389/fimmu.2026.1884482

**Published:** 2026-06-30

**Authors:** Haoyun Xia, Tianxing Guo, Nan Zhang, Dajia Lin, Guodong Guo, Yue Tian, Chenshen Huang, Shen Guan, Fangqin Xue, Liqi Chen

**Affiliations:** 1 Shengli Clinical Medical College of Fujian Medical University, Fujian Provincial Hospital, Fuzhou, China; 2 College of Life Science and Technology, Huazhong University of Science and Technology, Wuhan, China; 3 Fuzhou University Affiliated Provincial Hospital, School of Medicine, Fuzhou University, Fuzhou, China; 4 Fujian Medical University Union Hospital, Fuzhou, China; 5 Department of Colorectal Surgery, Clinical Oncology School of Fujian Medical University, Fujian Cancer Hospital, Fuzhou, China

**Keywords:** artificial intelligence, gastric cancer, immune evasion, machine learning (ML), NMF (nonnegative matrix factorization), RNASE1

## Abstract

Although immune checkpoint blockade (ICB) has changed treatment strategies for gastric adenocarcinoma (STAD), many patients show primary resistance. Tumor-intrinsic transcriptional heterogeneity may contribute to immune escape, but the malignant-cell programs and candidate biomarkers linked to this process remain incompletely defined. Here, we integrated single-cell transcriptomic profiles of 162,116 cells from 35 STAD samples with multiple bulk transcriptomic cohorts to characterize malignant-cell regulatory programs. Non-negative matrix factorization identified meta-program 2 (MP2), a malignant-cell program closely associated with immune-escape signatures. Through integrated co-expression analysis and machine-learning-based prioritization, RNASE1 was identified as an MP2-related candidate biomarker. High RNASE1 expression was associated with poor survival, higher immune and stromal scores, increased immune-checkpoint expression, and enrichment of ICB-response-related transcriptomic signatures. *In vitro* RNASE1 knockdown reduced STAD cell proliferation, migration, and invasion, supporting a functional contribution to malignant cellular phenotypes. Together, these findings suggest that RNASE1 is an immune-evasion-associated biomarker with potential value for prognosis and ICB-response stratification in STAD, while prospective clinical and *in vivo* validation remain necessary.

## Introduction

Gastric adenocarcinoma (STAD) ranks as the fifth most prevalent malignant neoplasm and the fourth leading cancer-related cause of mortality worldwide, accounting for more than 1 million new diagnoses and 769,000 deaths each year (1). Despite significant advances in surgical resection and targeted agents, the 5-year overall survival (OS) rate for advanced-stage STAD remains persistently low. Although immune checkpoint blockade (ICB) has improved treatment options for several solid tumors, only a subset of STAD patients derive durable clinical benefit, with objective response rates (ORRs) of less than 20% (2). Primary and acquired resistance therefore remain important clinical challenges, highlighting the need to identify molecular features associated with immune evasion and treatment response.

Intratumoral heterogeneity (ITH) is a key feature of cancer and is closely associated with tumor progression, therapeutic resistance, and immune escape (3). Recent breakthroughs in single-cell RNA sequencing (scRNA-seq) technology have enabled high-resolution dissection of the STAD tumor microenvironment (TME), revealing the complexity of immune and stromal cell subsets (4). However, many previous studies have mainly characterized non-malignant compartments. The transcriptional heterogeneity of malignant cells, and how tumor-intrinsic programs are associated with immune escape-related states, remains incompletely understood. Immune escape is a defining hallmark of cancer and the primary mechanism underlying ICB resistance (5). While previous investigations have identified numerous immune escape-related gene signatures using bulk transcriptomic data, these signatures cannot distinguish tumor-derived signals from TME-derived ones, limiting their clinical utility in tracking disease progression.

RNASE1 encodes a secreted ribonuclease involved in extracellular RNA metabolism and has been linked to endothelial activation, systemic inflammation, and tumor-associated immune regulation. Recent studies suggest that RNASE1 can promote malignant progression in selected solid tumors and may impair CD8+ T-cell function through immune-regulatory mechanisms. These observations provide a biological rationale for investigating RNASE1 in STAD, particularly in the context of tumor-intrinsic programs associated with immune escape. However, whether RNASE1 is connected to malignant-cell heterogeneity, prognosis, and ICB-related immune phenotypes in STAD remains unclear.

To investigate tumor-intrinsic immune-genetic programs in STAD, we integrated single-cell transcriptomic profiles of 162,116 cells from 35 STAD samples with multiple bulk transcriptomic cohorts. We mapped the cellular landscape, focused on malignant epithelial-cell heterogeneity, and identified an immune escape-associated meta-program (MP2). RNASE1 was then prioritized as an MP2-related candidate marker through integrated co-expression and machine-learning-based screening. We further evaluated its association with prognosis, immune features, ICB-response signatures, and malignant cellular phenotypes using *in vitro* knockdown assays. These analyses support RNASE1 as an immune-evasion-associated biomarker with potential value for prognostic and immunotherapy-related stratification in STAD.

The analytical workflow proceeded in four steps: first, scRNA-seq data were used to define malignant-cell states and identify an immune-escape-associated meta-program; second, single-cell and bulk co-expression networks were integrated to prioritize MP2-related candidate genes; third, RNASE1 was evaluated in relation to prognosis, immune infiltration, immunomodulatory features, and ICB-response signatures; and fourth, RNASE1 knockdown assays were used to assess its functional contribution to malignant cellular phenotypes.

## Materials and methods

### Study cohorts and data acquisition

Bulk transcriptomic matrices and matched clinical metadata were curated from three independent STAD cohorts: TCGA-STAD (6), GSE62254 (7), and GSE84437 (8). Samples with available expression data and survival information were retained for downstream analyses. For high-resolution cellular profiling, primary STAD scRNA-seq datasets were obtained from GEO accessions GSE163558 (9) and GSE183904 (10), encompassing 35 patient samples and 162,116 high-quality cells after quality control. Downstream scRNA-seq quality control, normalization, and dimensional reduction were executed utilizing the Seurat pipeline in R (11). Furthermore, a previously benchmarked immune escape (IE) transcriptional signature was adopted to facilitate subsequent cell-state scoring (12). For TCGA-STAD, normalized RNA-seq expression values were used for downstream analyses and log-transformed where appropriate. For GEO microarray cohorts, normalized expression matrices provided by GEO or the original studies were used, followed by gene-symbol annotation. For scRNA-seq datasets, Seurat-normalized expression values were used for visualization, clustering, and module scoring. To further evaluate the prognostic relevance of RNASE1 beyond STAD, we performed a pan-cancer survival analysis across 33 TCGA cancer types. For each cancer type, normalized RNASE1 expression and matched overall survival information were analyzed using univariate Cox proportional hazards regression.

### The scRNA-seq data processing

Cells with 200–6000 detected genes and less than 20% mitochondrial gene expression were retained. Gene expression matrices were normalized using Seurat NormalizeData, scaled using ScaleData, and integrated using the Seurat anchor-based integration workflow to reduce dataset-level batch effects before clustering and UMAP visualization.

### Malignant cell delineation via genomic copy number estimation

To segregate malignant populations from the non-malignant microenvironment, computational estimation of large-scale chromosomal copy number alterations was executed using the infercnv algorithm. Patient-matched normal epithelial cells served as the diploid calibration baseline. Epithelial subsets demonstrating extensive genomic instability, quantified by widespread chromosomal amplifications or deletions alongside statistically elevated aggregate CNV scores, were classified as the malignant compartment. The topological distribution of these annotated single-cell profiles was subsequently projected into a two-dimensional coordinate space utilizing UMAP embeddings.

### Computational extraction of malignant meta-programs

To characterize transcriptional programs within malignant cells, non-negative matrix factorization (NMF) was applied to normalized expression matrices using the GeneNMF toolkit (13). NMF ranks were evaluated by cophenetic correlation and residual error, and k = 8 was selected as the final rank because it provided stable decomposition of malignant-cell transcriptional programs. The resulting programs were defined as intratumoral heterogeneity-associated meta-programs (MPs). MP2 was defined as the NMF-derived malignant-cell meta-program showing the strongest concordance with the immune escape signature. In single-cell data, MP2 activity was quantified using AUCell, which estimates whether MP2 genes are enriched among the highly ranked genes in each cell.

### High-dimensional network construction and algorithmic feature selection

To delineate the regulatory networks governing the MP2 signature at single-cell resolution, we deployed the hdWGCNA framework (14). A scale-free topological matrix was engineered by applying a soft-thresholding parameter of 16. In parallel, bulk-level MP2 activity across the TCGA-STAD cohort was quantified via single-sample Gene Set Enrichment Analysis (ssGSEA). Subsequent macro-level co-expression modules were synthesized utilizing the traditional WGCNA algorithm (15), mathematically calibrated with a soft-thresholding power of 5 to enforce strict scale-free topology metrics. Finally, to isolate critical prognostic drivers from the resulting multidimensional feature space, consecutive algorithmic reduction was executed leveraging CoxBoost (16) alongside Random Survival Forest (RSF) (17) modeling.

### Immunological profiling and pathway deconvolution

To quantify the stromal-immune admixture within the tumor microenvironment, absolute tumor purity alongside discrete stromal and immune indices were computationally derived utilizing the ESTIMATE framework (18). Furthermore, the microenvironmental infiltration landscape was orthogonally mapped employing three distinct algorithms: MCPcounter (19), TIMER (22), and single-sample GSEA (ssGSEA) configured with Porpimol’s reference matrices (20). To evaluate the potential of RNASE1 in reflecting immunotherapeutic vulnerability, its expression dynamics were cross-examined against a comprehensive panel of nine transcriptomic proxies for ICB responsiveness. Rather than a monolithic metric, this panel incorporated effector and chemokine signals [CYT (21), IFNγ (22), Chemokines (23)], local inflammation profiles [Ayers expIS (22), T-cell-inflamed (22)], and multifactorial predictive models [Roh IS (24), Davoli IS (25), RIR (26), and ICBnetIS (27, 28)]. Finally, the downstream biological cascades correlated with RNASE1 were delineated via the Metascape analytical suite (29) coupled with Gene Ontology-driven GSEA.

### Cell culture and RNA interference

*In vitro* models of gastric adenocarcinoma, specifically the AGS and HGC-27 lineages, were procured from Cellverse Co., Ltd. Basal maintenance was performed utilizing Dulbecco’s Modified Eagle Medium (DMEM), which was enriched with a 10% volume fraction of fetal bovine serum (FBS) alongside a standard 1% penicillin-streptomycin antibiotic cocktail. The cellular microenvironment was strictly regulated within a humidified incubation chamber maintained at 37 °C and a 5% CO_2_ atmosphere. For loss-of-function investigations, transient transcript depletion was achieved via liposome-mediated interference. Sequence-specific small interfering RNAs directed against the human RNASE1 transcript (si-RNASE1), parallel to a randomized non-targeting control sequence (si-NC), were delivered into the cellular hosts utilizing the Lipofectamine 3000 reagent (Invitrogen) under optimized parameters. All subsequent phenotypic and functional evaluations were uniformly executed at a 48-hour temporal checkpoint post-transfection.

### *In vitro* phenotypic and functional evaluations

To visualize target protein localization, immunocytochemical labeling was conducted adapting the methodological framework outlined previously (30). *De novo* DNA synthesis, serving as a direct metric for proliferative vigor, was quantified via 5-ethynyl-2’-deoxyuridine (EdU) integration under optimized analytical conditions. The directional motility and extracellular matrix penetration capacities of the engineered STAD cohorts were evaluated deploying bicameral Transwell permeable supports (8-μm porosity, Corning). For invasion dynamics, the apical interface was pre-polymerized with a Matrigel matrix barrier, a step deliberately omitted for evaluating pure chemotactic migration. Following a 48-hour post-transfection stabilization phase, cellular suspensions formulated in serum-deprived basal media were inoculated into the upper compartments. Concurrently, a 20% FBS concentration gradient was established in the basal chambers to serve as the chemotactic stimulus. After a 24-hour dissemination window, the sub-membrane transmigrant populations were immobilized utilizing a 4% paraformaldehyde fixative, optically contrasted via 0.1% crystal violet staining, and subjected to quantitative microscopic enumeration.

### Statistical analysis

All statistical analyses were performed in R. Wilcoxon rank-sum tests were used for two-group comparisons, log-rank tests were used for Kaplan-Meier survival analyses, and Spearman correlation was used for association analyses unless otherwise stated. P < 0.05 was considered statistically significant. Unless otherwise specified, reported P values are nominal P values.

## Results

### Single-cell transcriptomic mapping defines the major cellular compartments of the STAD microenvironment

**Figure 1:**
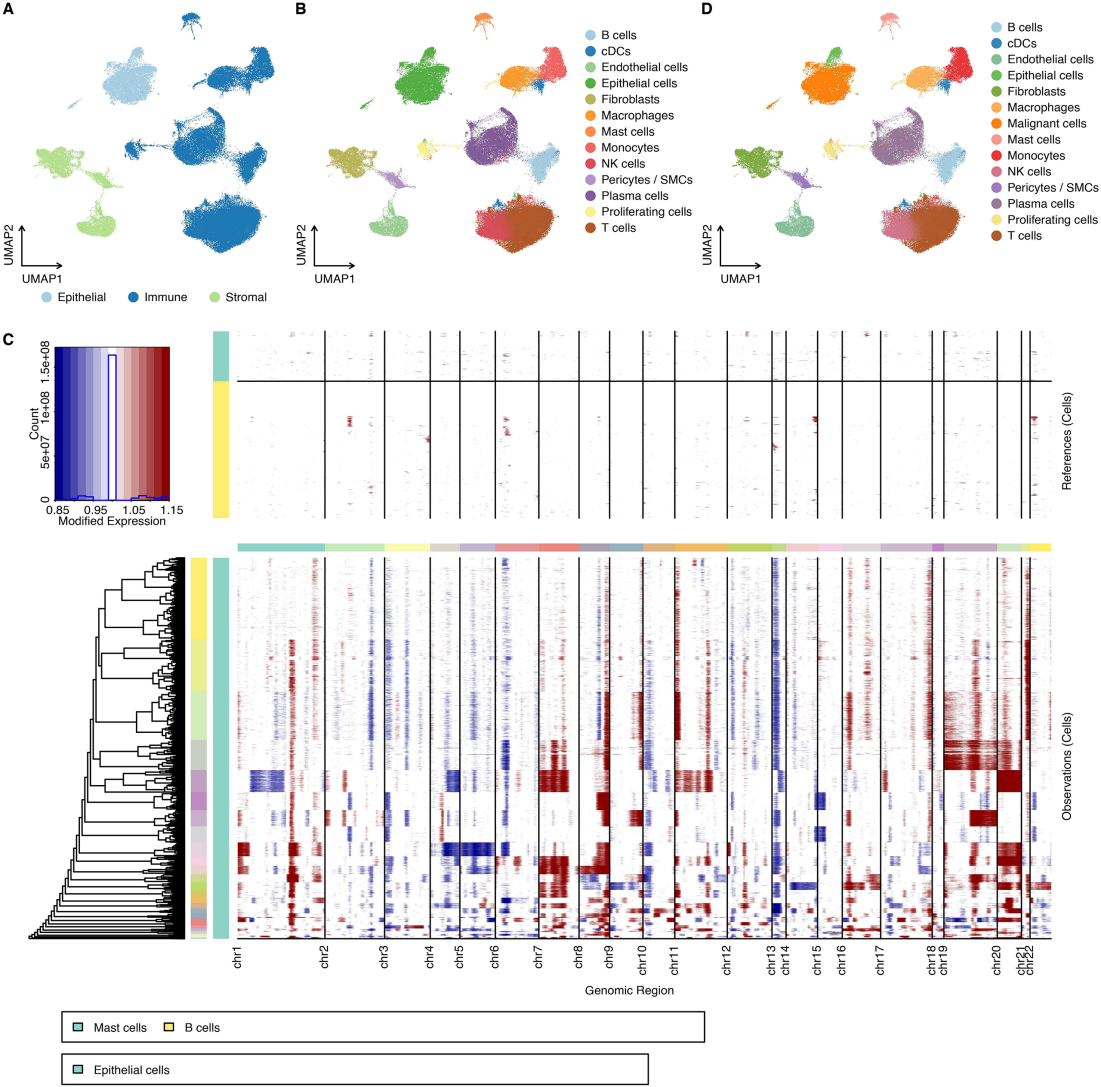
High-dimensional single-cell architecture of the STAD microenvironment **(A)** Macro-level UMAP projection partitioning the primary TME compartments. **(B)** High-resolution topological embedding of 14 discrete cellular subpopulations. **(C)** Heatmap quantifying inferred genomic copy number variations (CNVs), utilizing normal epithelial clusters as the diploid calibration standard to empirically delineate the malignant fraction. **(D)** Comprehensive UMAP spatial distribution of all annotated cellular subsets.

To characterize the cellular composition of the STAD tumor microenvironment (TME), we integrated single-cell transcriptomic profiles from 35 patient samples (GEO: GSE163558 and GSE183904). After quality control, 162,116 cells were retained for clustering and annotation. The major compartments included epithelial, immune, and stromal lineages (**Figure 1A**). Further annotation identified 14 cell populations, including B cells, conventional dendritic cells (cDCs), endothelial cells, epithelial cells, fibroblasts, macrophages, malignant cells, mast cells, monocytes, natural killer (NK) cells, pericytes/smooth muscle cells (SMCs), plasma cells, proliferating cells, and T cells (**Figures 1B, D**).

To distinguish malignant epithelial cells from non-malignant epithelial cells, inferCNV was used to estimate large-scale copy-number patterns. Using normal epithelial clusters as the reference baseline, we identified epithelial cells with broad chromosomal gains or losses and higher CNV scores, which were annotated as malignant cells (**Figure 1C**).

**Figure 2:**
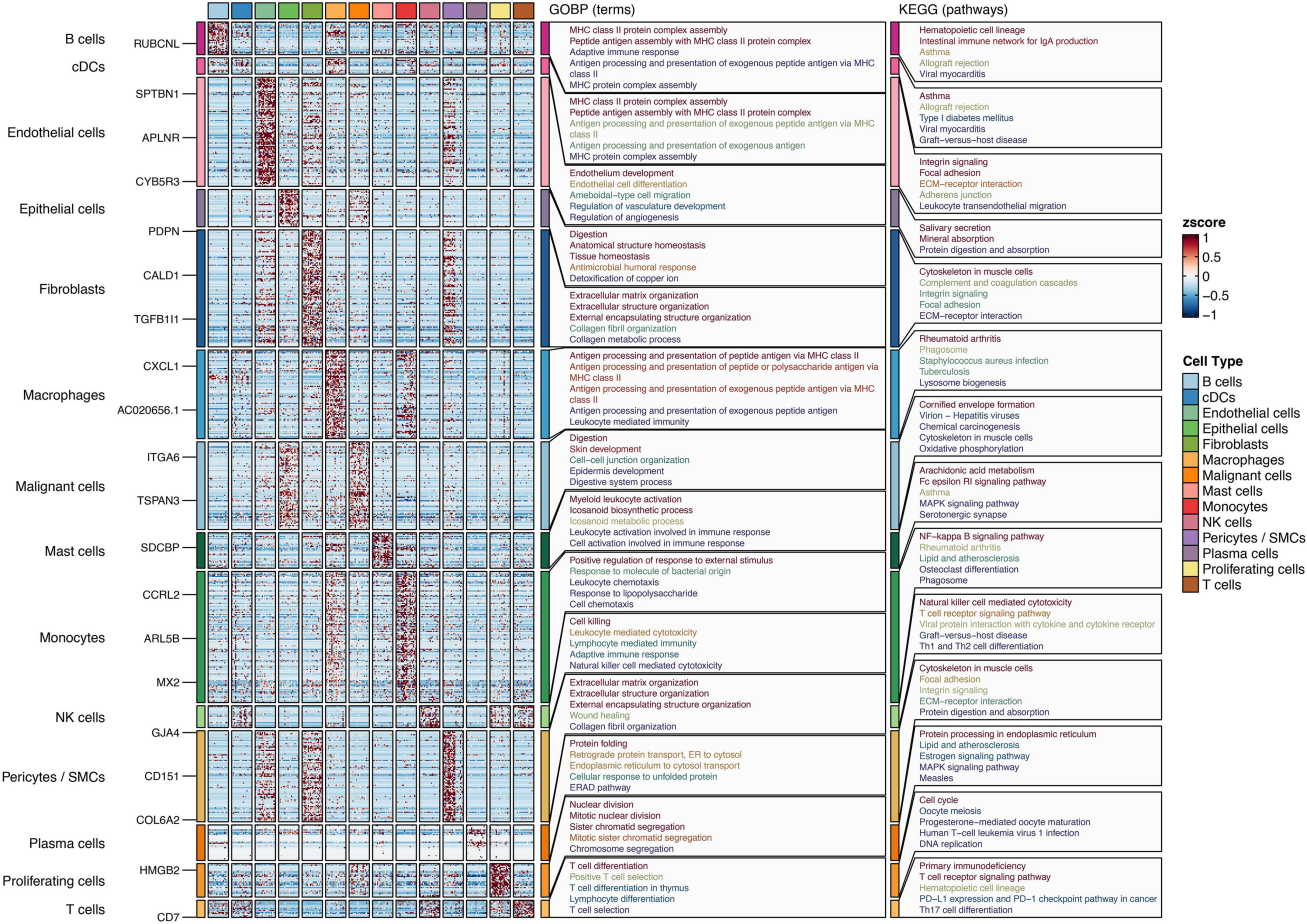
Transcriptional identity mapping and functional deconvolution of TME subpopulations Expression matrix of canonical lineage-specific identifiers across the 14 microenvironmental subsets, aligned with corresponding biological trajectory enrichments derived via GO and KEGG algorithmic frameworks.

The identities of the 14 cell populations were supported by canonical lineage markers, including CD7 for T cells, RUBCNL for B cells, CXCL1 for macrophages, and ITGA6 for malignant cells (**Figure 2**). GO and KEGG analyses further showed cell-type-related pathway patterns: T cell clusters were enriched for cytotoxic effector programs, endothelial cells were enriched for angiogenesis-related pathways, and malignant cells showed enrichment of cell-cycle, epithelial-mesenchymal transition, and invasion-associated programs (**Figure 2**). After defining the major cellular compartments, we next focused on malignant epithelial cells, because tumor-intrinsic programs are most directly related to immune escape and therapeutic resistance.

### NMF analysis identifies a malignant-cell meta-program associated with immune escape

**Figure 3:**
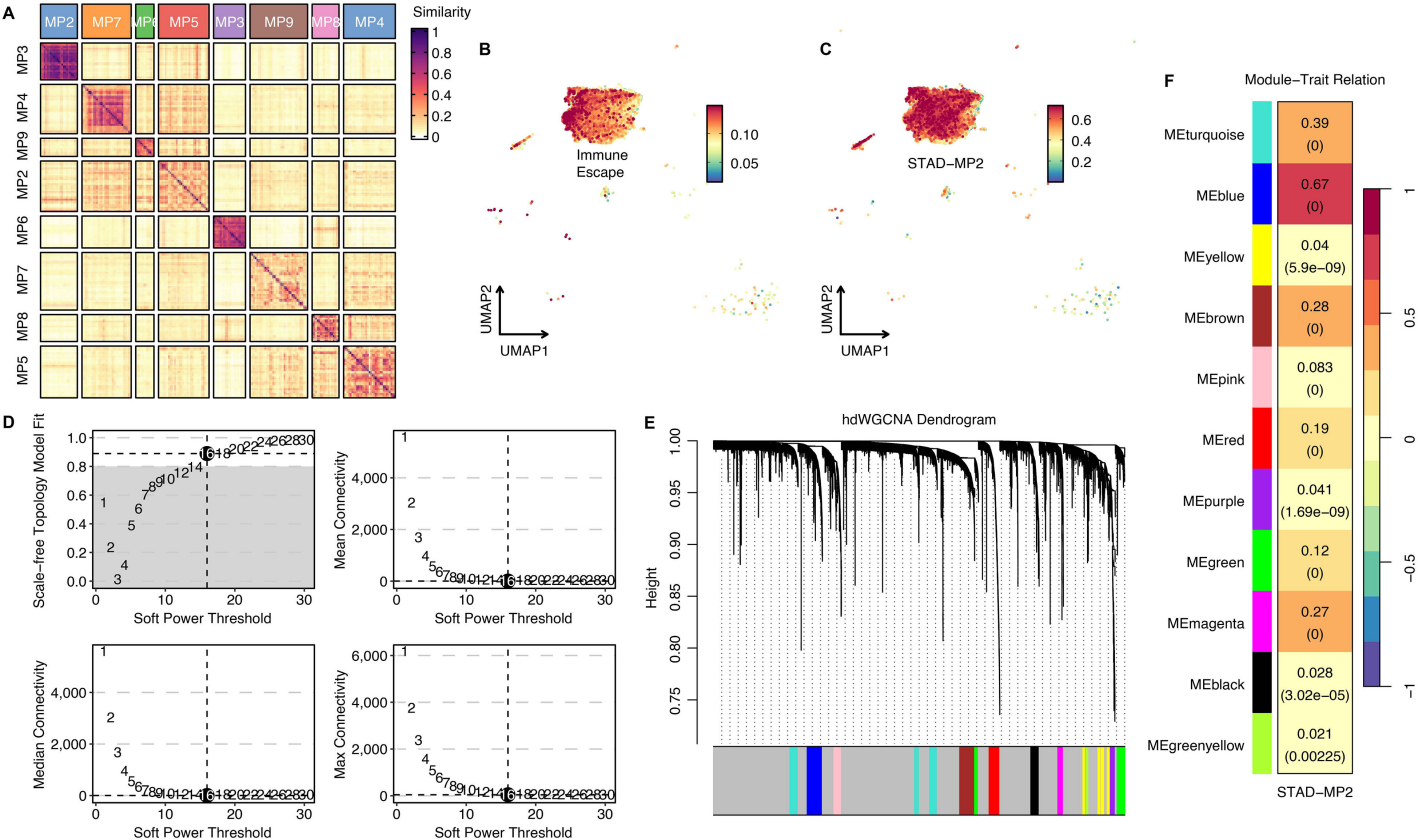
Single-cell algorithmic extraction of malignant meta-programs and the immune-escape regulatory network **(A)** Hierarchical clustering and Jaccard similarity matrix of 8 discrete transcriptional states extracted via non-negative matrix factorization (NMF). **(B, C)** UMAP spatial projections mapping the distribution of **(B)** baseline immune escape (IE) and **(C)** MP2 module activities across the malignant continuum. **(D)** Parameter optimization for hdWGCNA, depicting the scale-free topology fit against soft-thresholding connectivity. **(E)** Algorithmic segregation of single-cell transcriptomes into co-expression modules. **(F)** Correlational matrix linking hdWGCNA-derived genetic modules to the MP2 signature, with statistical alignments annotated.

To decode the functional diversity within the isolated malignant compartment, non-negative matrix factorization (NMF) was deployed across the normalized expression matrices via the GeneNMF algorithm. Mathematical convergence dictated an optimal factorization rank of k=8, anchored by the peak cophenetic correlation coefficient and the minimization of the residual sum of squares. This matrix decomposition successfully partitioned the malignant population into 8 discrete, recurrent transcriptional meta-programs (MPs) (**Figure 3A**).

To evaluate the immunological relevance of these defined states, module activity for a validated immune escape (IE) signature was quantified at single-cell resolution utilizing the AUCell framework. Topological projection of these scores demonstrated marked intra-lineage variability across the malignant compartment (**Figure 3B**). Quantitative spatial alignment revealed a near-congruent distribution between the MP2 module activity and the IE signature scores (**Figure 3C**). This spatial overlap was mathematically corroborated by Jaccard similarity indexing, which ranked MP2 as having the highest topological concordance with the IE signature among all extracted programs. These empirical data indicate that MP2 represents the primary transcriptional network orchestrating immune evasion within STAD malignant cells.

To identify co-expression modules associated with the MP2 state, hdWGCNA was applied to malignant cells. A scale-free network was constructed using a soft-thresholding power of 16 (**Figure 3D**), and 12 co-expression modules were identified (**Figure 3E**). The blue module showed the strongest correlation with MP2 activity (r=0.67, P<0.05; **Figure 3F**) and was therefore selected for downstream candidate-gene prioritization. Having identified MP2 as the malignant-cell program most closely linked to immune escape at single-cell resolution, we next asked whether this co-expression pattern was reflected in bulk clinical cohorts.

### Bulk-cohort assessment of the MP2-associated co-expression network in STAD

**Figure 4:**
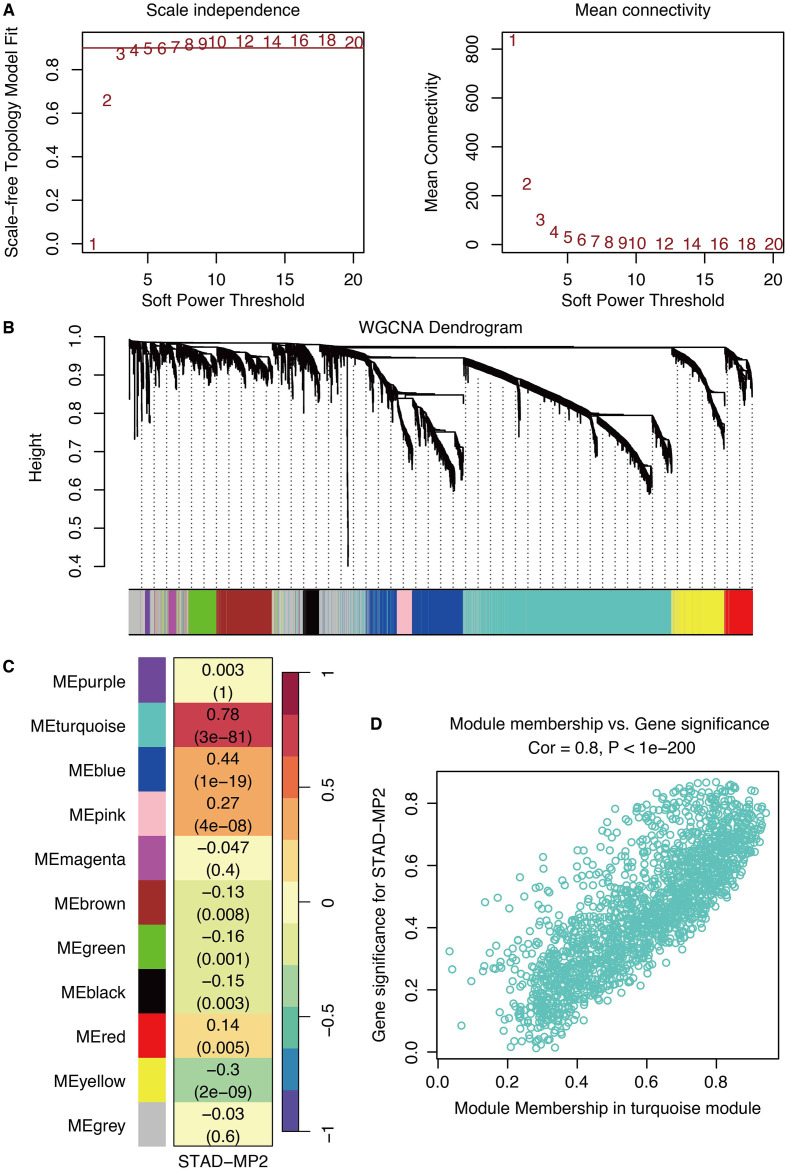
Macro-level structural validation of the MP2-coupled genetic network **(A)** Topological calibration metrics for bulk WGCNA network synthesis. **(B)** Distribution profile of tissue-level co-expression modules. **(C)** Statistical correlation matrix linking bulk-derived modules to the overarching MP2 state. **(D)** Scatter plot validating the stringent positive correlation between gene significance (GS) for the MP2 network and module membership (MM) within the apex (turquoise) cluster.

To examine whether MP2-associated co-expression patterns were reflected in bulk clinical data, MP2 activity was quantified in the TCGA-STAD cohort using ssGSEA. WGCNA was then performed with a soft-thresholding power of 5 (**Figure 4A**), identifying 12 co-expression modules (**Figure 4B**). The turquoise module showed the strongest correlation with MP2 activity (r=0.78, P<0.05; **Figure 4C**). Gene significance for MP2 was also positively correlated with module membership within this module (r=0.8, P<0.05; **Figure 4D**). These results indicate that MP2-associated co-expression patterns observed at single-cell resolution were also reflected in the TCGA-STAD bulk cohort. The concordance between single-cell and bulk networks provided a narrowed candidate space for prioritizing clinically relevant MP2-associated genes.

### Multi-dimensional algorithmic screening prioritizes RNASE1 as a prognosis-associated candidate marker

**Figure 5:**
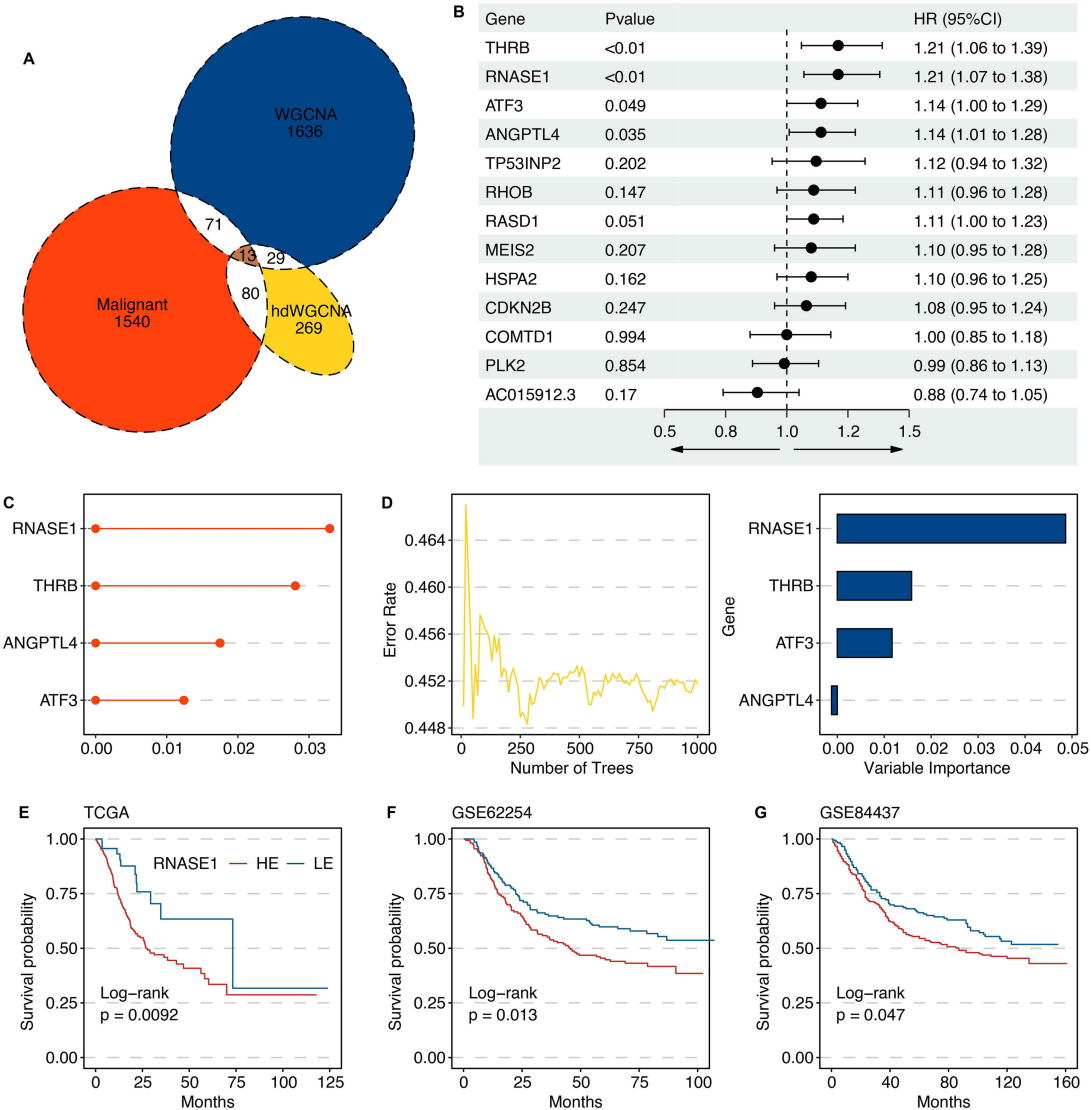
Multi-dimensional intersection isolates RNASE1 as an independent prognostic determinant **(A)** Venn diagram illustrating the convergence of candidates from hdWGCNA (single-cell), WGCNA (bulk), and differential expression pipelines. **(B)** Univariate Cox proportional hazards modeling of the 13 overlapping transcripts. **(C, D)** Algorithmic dimensionality reduction prioritizing candidate drivers via **(C)** CoxBoost and **(D)** Random Survival Forest (RSF) machine learning models. **(E–G)** Kaplan-Meier survival trajectories stratifying overall survival based on RNASE1 expression across the **(E)** TCGA-STAD, **(F)** GSE62254, and **(G)** GSE84437 cohorts.

To distill the apex functional regulators governing the MP2 program, an integrative intersection analysis was executed utilizing three orthogonal gene sets: (1) the hdWGCNA-derived blue module (single-cell peak association), (2) the WGCNA-derived turquoise module (bulk-level peak association), and (3) transcripts statistically overexpressed in the malignant epithelium relative to the microenvironment. This convergence isolated 13 high-priority candidates (**Figure 5A**). Subsequent univariate Cox regression modeling within the TCGA-STAD matrix identified four distinct transcripts as independent adverse prognostic determinants: THRB (HR = 1.21, P<0.01), RNASE1 (HR = 1.21, P<0.01), ATF3 (HR = 1.14, P = 0.049), and ANGPTL4 (HR = 1.14, P = 0.035) (**Figure 5B**). To mathematically isolate the singular core driver, consecutive dimensionality reduction was enacted via CoxBoost and Random Survival Forest (RSF) machine learning models. Both algorithms independently ranked RNASE1 with the peak feature importance (**Figures 5C, D**). The prognostic severity of RNASE1 was corroborated across three independent validation matrices (TCGA-STAD, GSE62254, and GSE84437). Survival probability assessments confirmed significant overall survival detriments in high-RNASE1 expression subsets across all cohorts (TCGA-STAD: P = 0.0092; GSE62254: P = 0.013; GSE84437: P = 0.047) (**Figures 5E–G**). To place this STAD-centered finding in a broader cancer context, we further performed a pan-cancer univariate Cox analysis of RNASE1 across 33 TCGA cancer types. RNASE1 showed significant survival associations in 15 tumor types, including STAD (**Supplementary Figure 1**).

### RNASE1 knockdown reduces malignant cellular phenotypes *in vitro*

**Figure 6:**
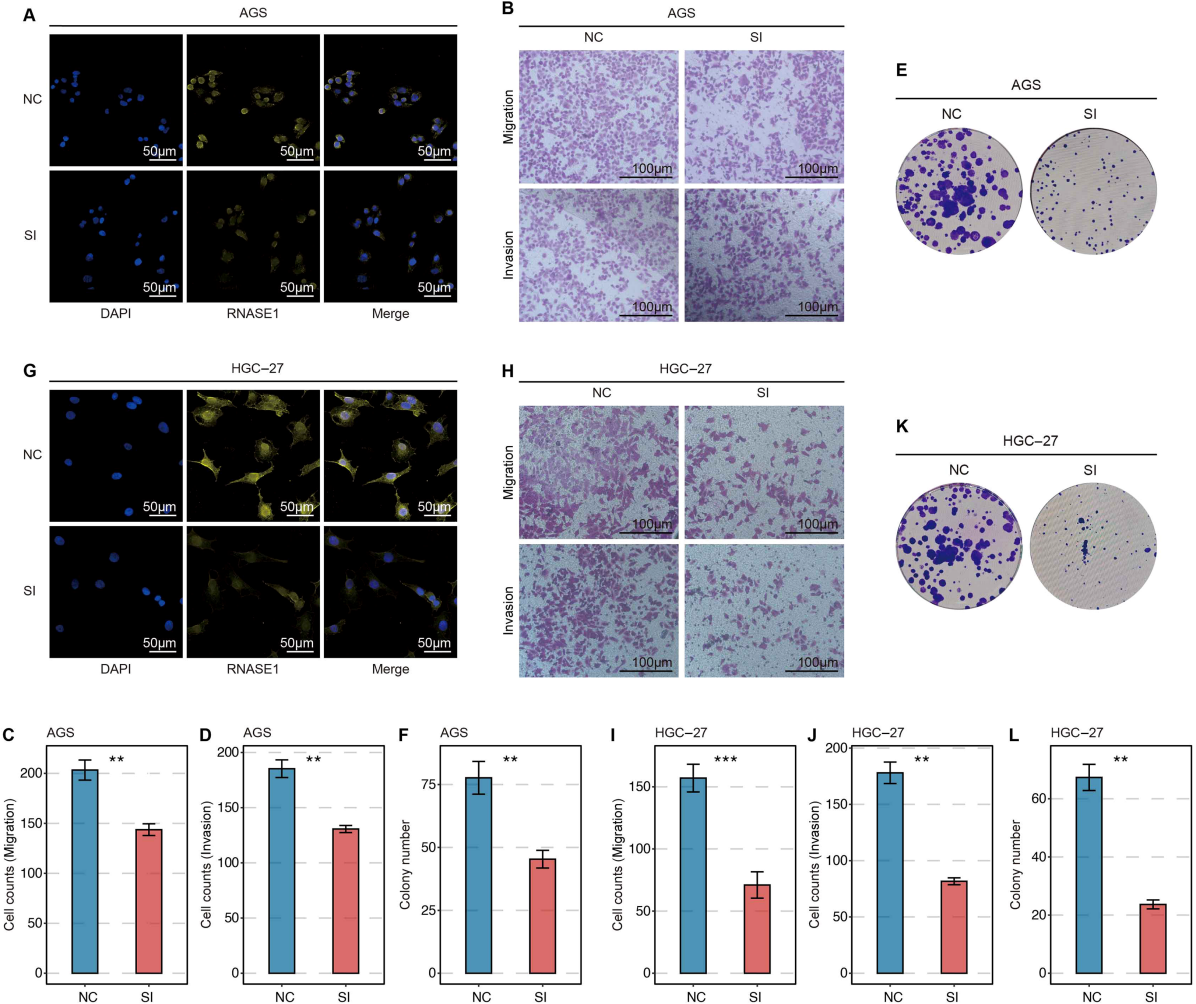
RNASE1 transcript depletion attenuates malignant phenotypic expansion and spatial invasion *in vitro* **(A, G)** Microscopic validation of targeted RNASE1 protein suppression via immunocytochemical labeling in AGS **(A)** and HGC-27 **(G)** models. **(B–D, H–J)** Bicameral Transwell evaluations quantifying chemotactic migration and matrix-degrading invasion capabilities following RNASE1 interference in AGS **(B–D)** and HGC-27 **(H–J)** lineages. **(E, F, K, L)** Clonogenic survival dynamics demonstrating restricted colony formation capacities in RNASE1-depleted AGS **(E, F)** and HGC-27 **(K, L)** cells. **P < 0.01, ***P < 0.001.

**Figure 7:**
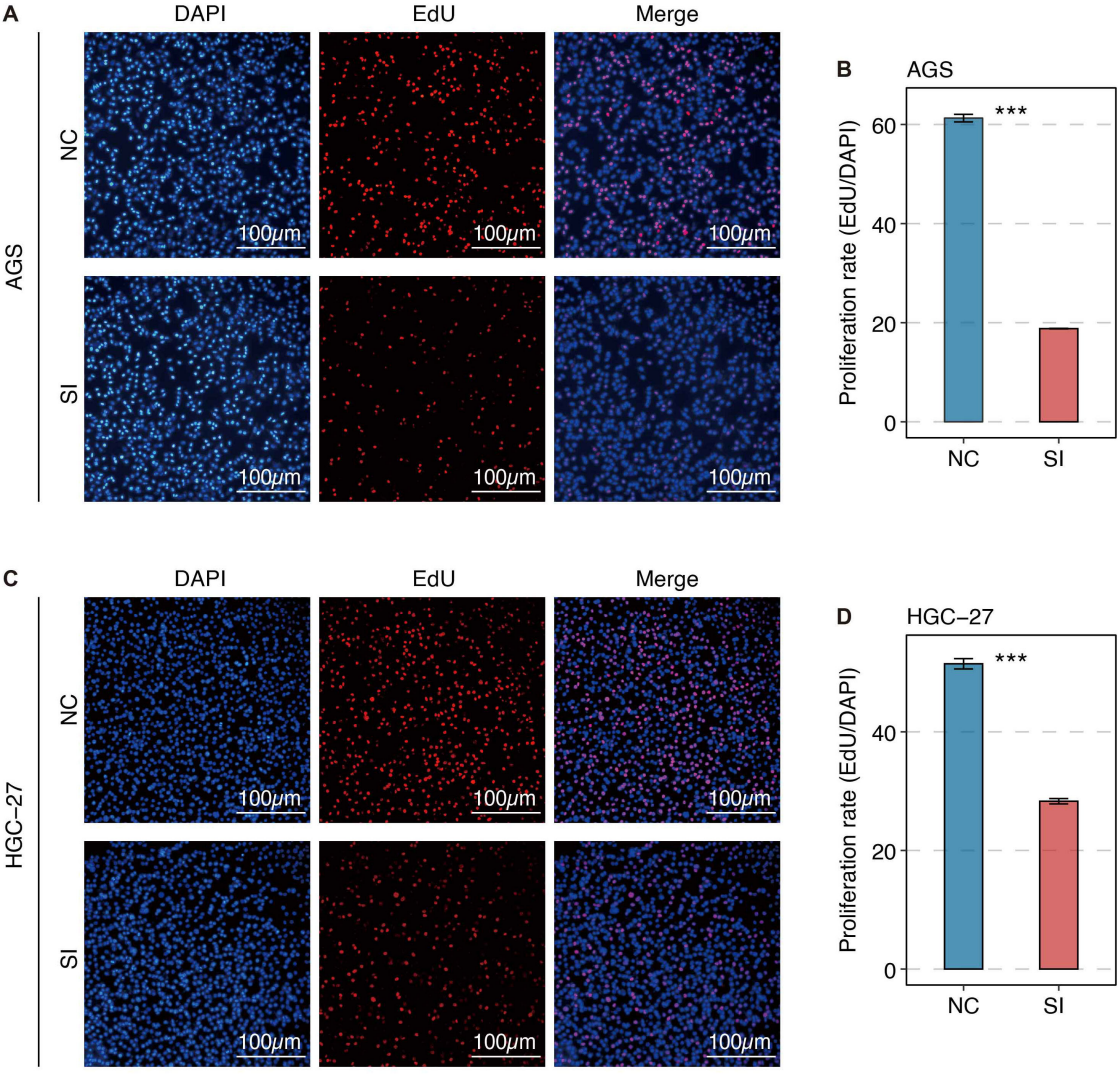
RNASE1 suppression restricts *de novo* DNA synthesis and proliferative vigor in gastric adenocarcinoma cells **(A–D)** Fluorescence-based EdU incorporation metrics and corresponding quantitative analysis comparing the actively dividing fractions between control and si-RNASE1 groups in AGS **(A, B)** and HGC-27 **(C, D)** models. ***P < 0.001.

To evaluate the functional consequence of RNASE1 during disease progression, sequence-specific transient transcript depletion (si-RNASE1) was executed in two STAD *in vitro* models (AGS and HGC-27). Subcellular protein depletion was microscopically verified via immunocytochemical labeling compared to randomized controls (**Figures 6A, G**). Phenotypic tracing demonstrated that RNASE1 suppression strictly curtailed basal clonogenic survival in both AGS (**Figures 6E, F**) and HGC-27 lineages (**Figures 6K, L**). Furthermore, chemotaxis and extracellular matrix degradation assays established that RNASE1 knockdown quantitatively impedes directional migration and structural invasion dynamics (**Figures 6B–D, H–J**). Finally, evaluating *de novo* DNA synthesis via EdU incorporation confirmed a statistically significant reduction in the actively proliferating fraction of RNASE1-depleted cells (**Figures 7A–D**). Collectively, these experimental models dictate that RNASE1 acts as a direct accelerator of malignant expansion and tissue invasion.

### RNASE1 expression is associated with an immune-infiltrated and checkpoint-enriched microenvironment

**Figure 8:**
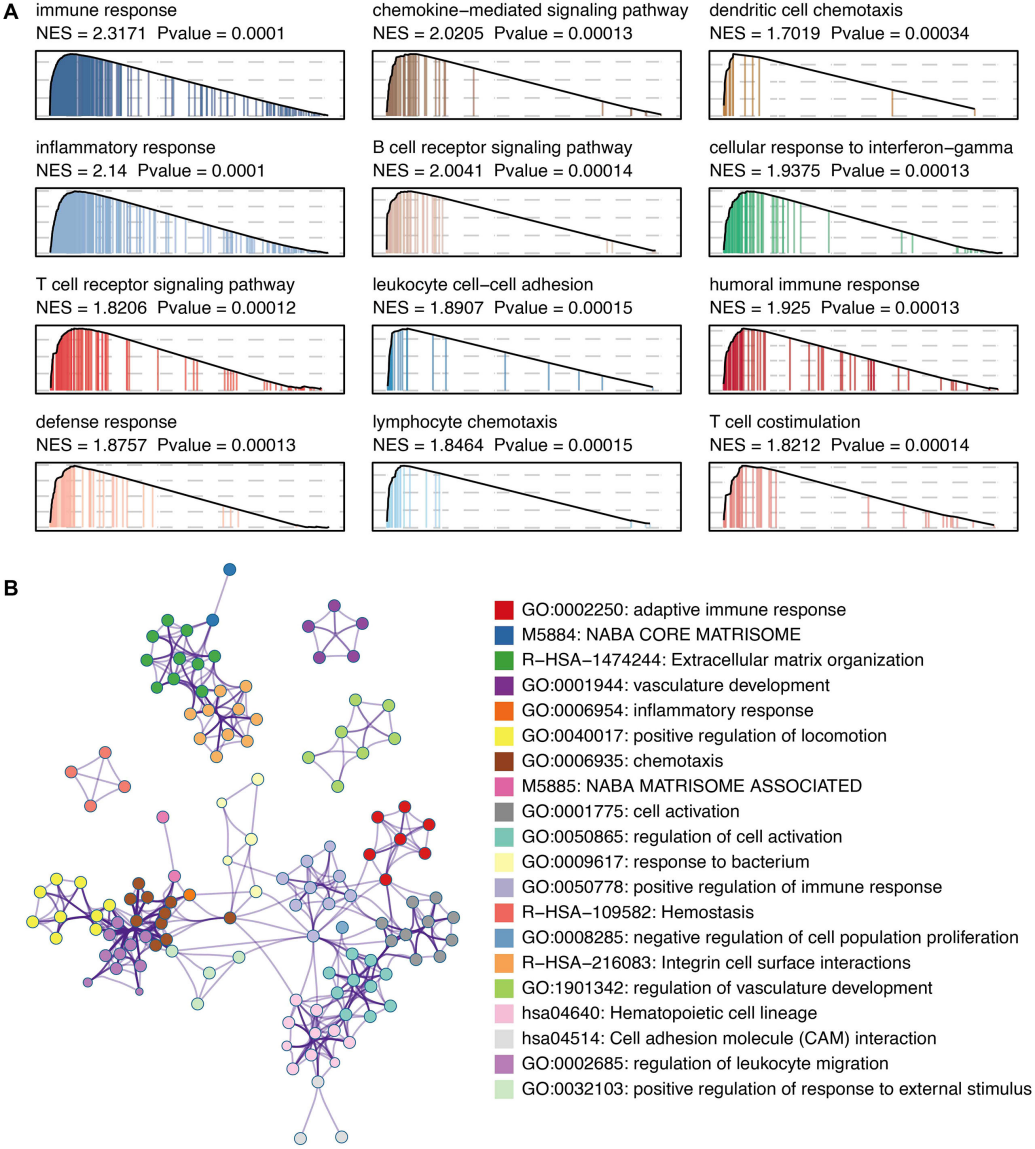
Biological cascade mapping and pathway deconvolution associated with RNASE1 activity **(A)** Gene Set Enrichment Analysis (GSEA) detailing the primary immunological and chemotactic cascades upregulated in the high-RNASE1 STAD subset. **(B)** Topological functional network of RNASE1-correlated transcripts synthesized via the Metascape analytical suite.

**Figure 9:**
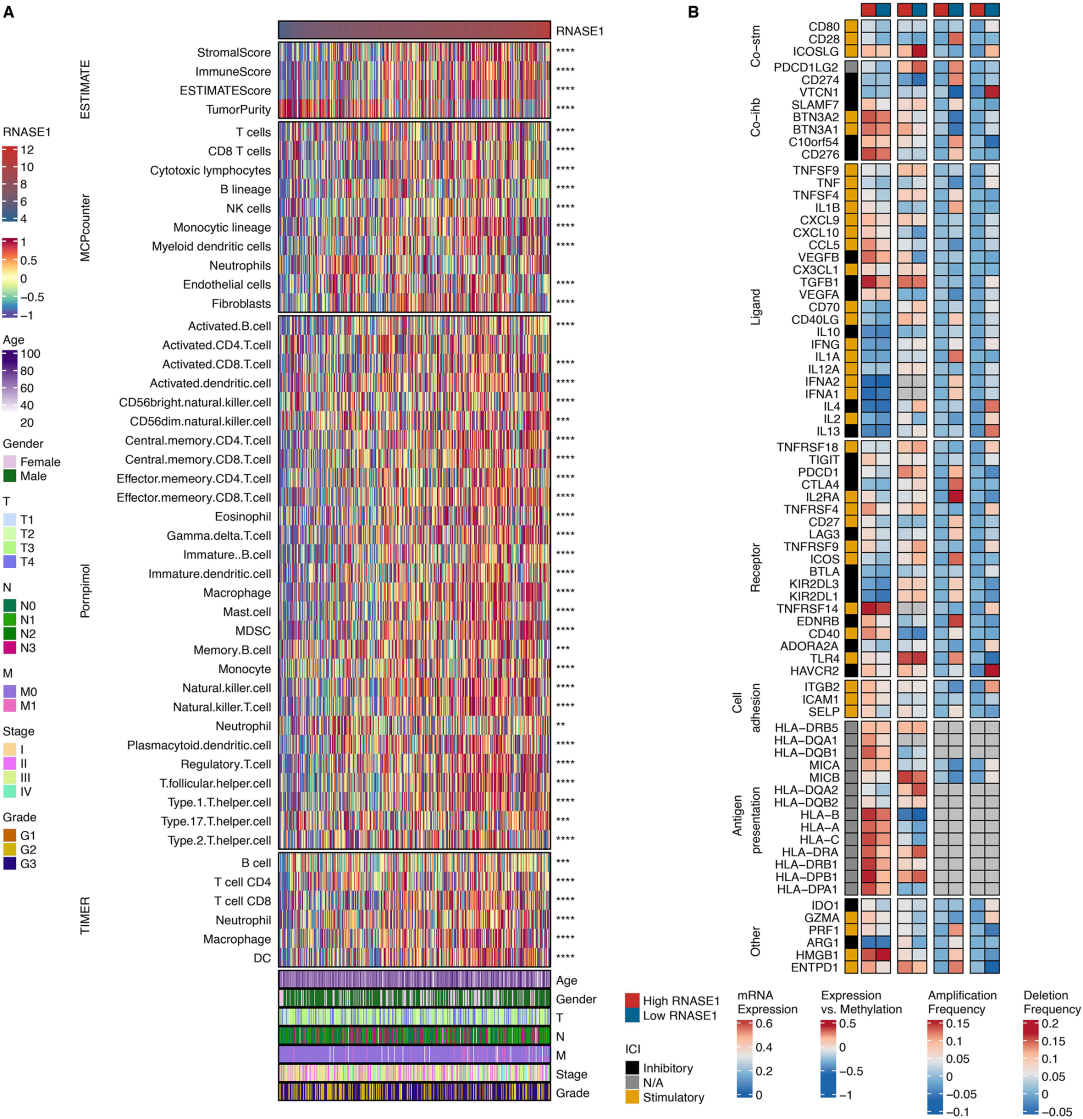
RNASE1 expression dictates an immunosuppressive microenvironmental landscape **(A)** Correlational matrix cross-examining RNASE1 levels with ESTIMATE-derived structural indices and cellular infiltration abundances inferred via MCPcounter, signature-based ssGSEA, and TIMER algorithms. **(B)** Heatmap profiling the stringent positive alignment between RNASE1 and 7 distinct classifications of immunomodulatory molecules, highlighting the coordinated upregulation of co-inhibitory checkpoints. **P < 0.01, ***P < 0.001, ****P < 0.0001.

To elucidate the specific immune-genetic dynamics modulated by RNASE1, Gene Ontology-driven GSEA was applied to the TCGA-STAD dataset. This unmasked a distinct correlation between elevated RNASE1 expression and a spectrum of immunological cascades, including leukocyte chemotaxis, and T/B cell receptor signaling (NES>1.7, P<0.001) (**Figure 8A**). Network synthesis via Metascape corroborated this functional alignment toward adaptive immunity and matrix organization (**Figure 8B**). Microenvironmental deconvolution, integrating ESTIMATE, MCPcounter, TIMER, and signature-based ssGSEA algorithms, demonstrated that RNASE1 activity was tightly coupled to elevated stromal and immune scores, concurrent with suppressed overall tumor purity (**Figure 9A**). Critically, while RNASE1 positively correlated with extensive infiltration of cytotoxic lymphocytes, dendritic cells, and macrophages, this influx was paralleled by a massive upregulation across 7 classes of immunomodulators. Most notably, this included critical co-inhibitory checkpoints such as PDCD1, CD274, CTLA4, LAG3, and TIGIT (**Figure 9B**). This specific compositional signature defines an “immune-excluded” microenvironment, mechanistically linking RNASE1 to active immune evasion despite high local inflammation.

### RNASE1 expression is associated with checkpoint blockade response patterns across public ICB cohorts

**Figure 10:**
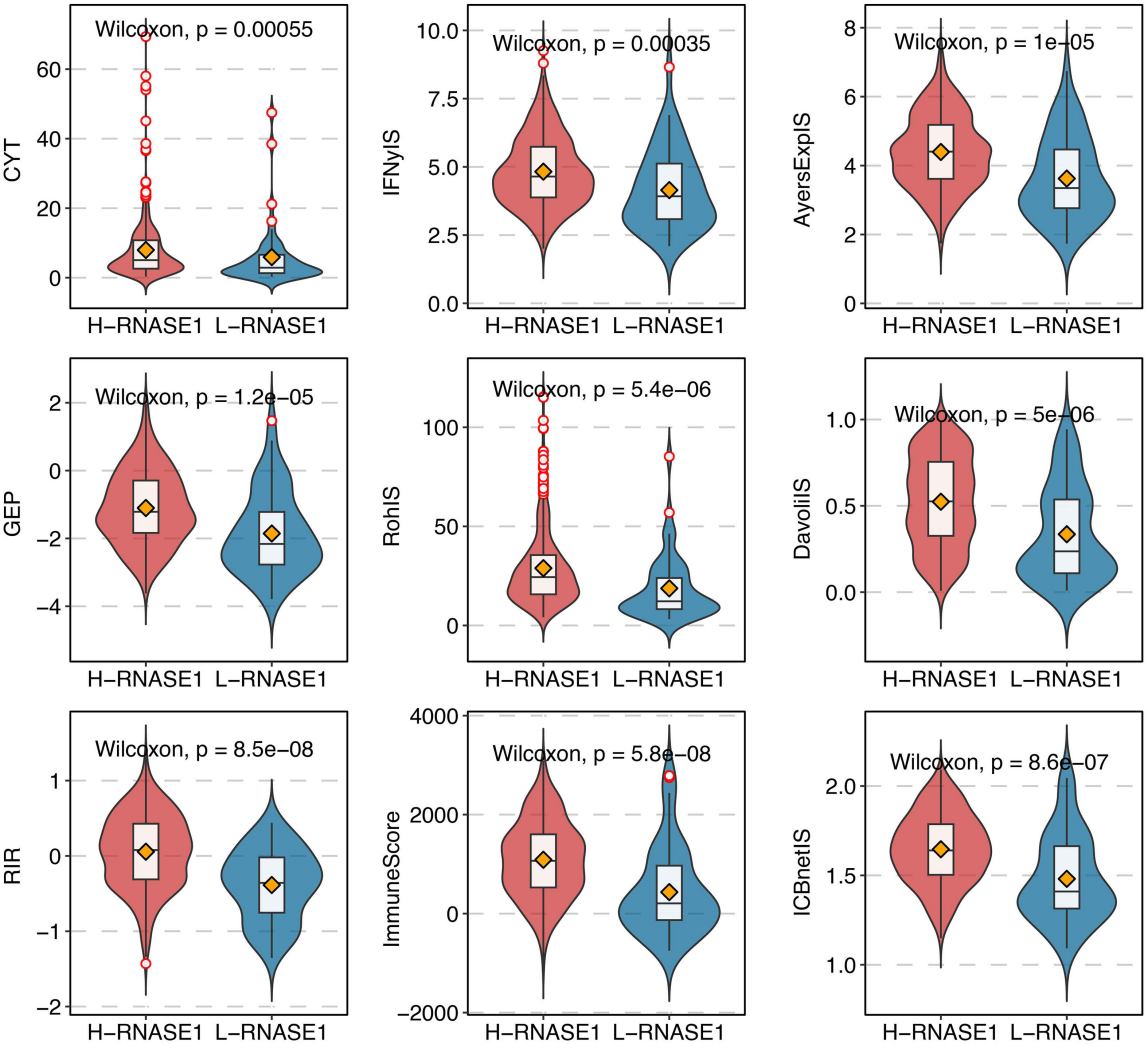
Alignment of RNASE1 with established immunotherapeutic response signatures Comparative quantification of 9 benchmarked transcriptomic ICB-response profiles, demonstrating significantly elevated enrichment scores within the high-RNASE1 STAD subpopulation.

**Figure 11:**
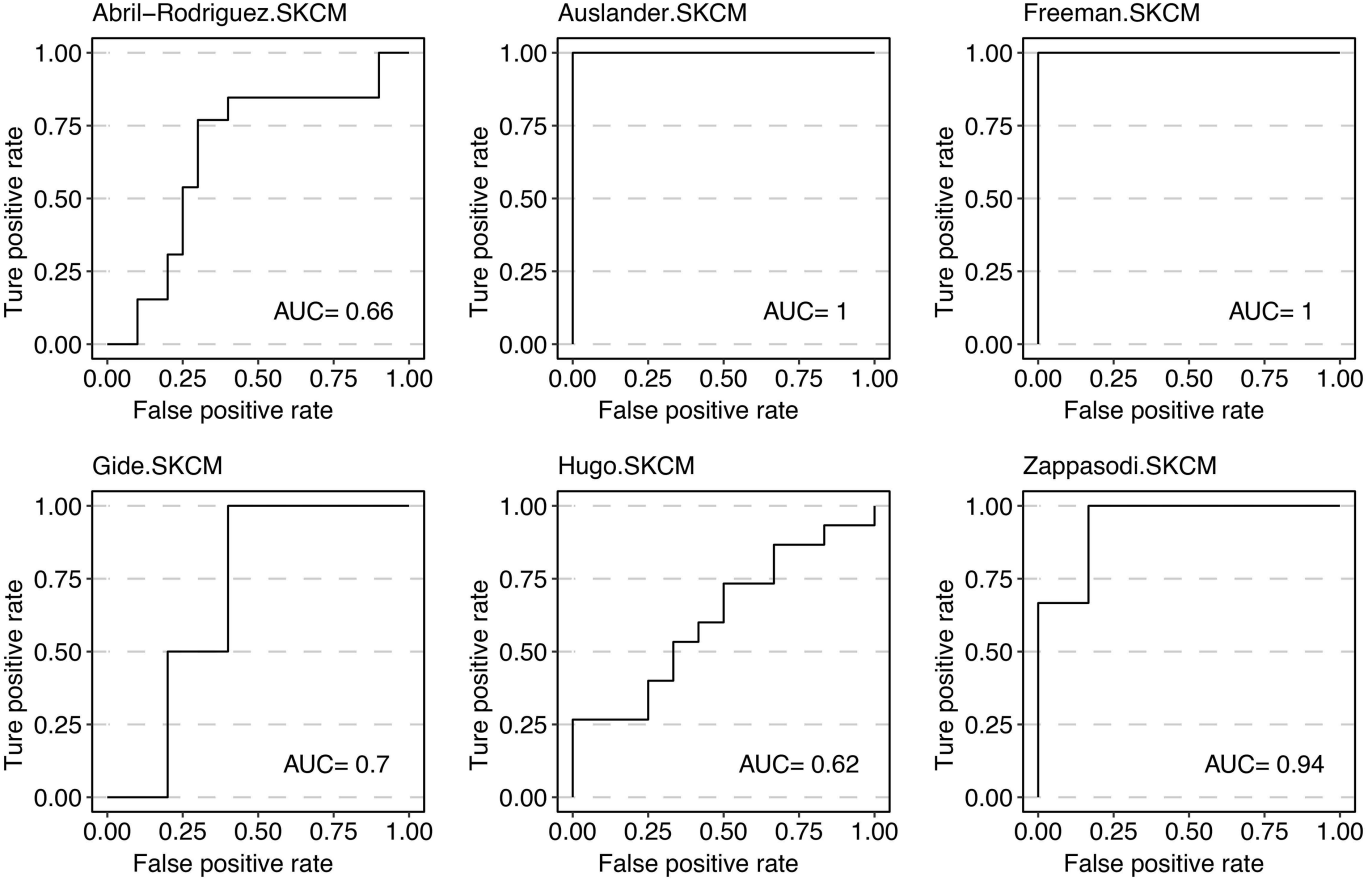
Cross-cohort predictive efficacy of RNASE1 for checkpoint blockade responsiveness Receiver operating characteristic (ROC) evaluations determining the discriminatory capability of RNASE1 expression for clinical outcomes across six independent ICB-treated patient matrices.

Driven by its central role in coordinating the immunosuppressive niche, the capacity of RNASE1 to guide therapeutic strategies was evaluated. Initial expression dynamics were cross-examined against 9 independent, well-validated transcriptomic proxies for ICB responsiveness. Enrichment scores for the entire panel (including CYT, IFNγ, Ayers expIS, and T-cell-inflamed signatures) were significantly elevated within the high-RNASE1 cohort (Wilcoxon P<0.001) (**Figure 10**). The predictive threshold of RNASE1 was subsequently stress-tested across 6 independent ICB-treated clinical cohorts. Receiver operating characteristic (ROC) evaluations confirmed significant discriminatory capability for clinical response, yielding area under the curve (AUC) metrics ranging from 0.6 to 1.0 (e.g., Auslander.SKCM AUC = 1.0; Zappasodi.SKCM AUC = 0.94) (**Figure 11**). These data formally establish RNASE1 as an objective, cross-tumor metric for determining patient suitability for immune checkpoint blockade.

## Discussion

The clinical translation of immune checkpoint blockade (ICB) in gastric adenocarcinoma (STAD) is fundamentally bottlenecked by primary resistance. While prior single-cell landscapes mapped non-malignant compartments (10, 31), the tumor-intrinsic immune-genetic dynamics driving this resistance have remained uncharacterized. By establishing a computational framework across 162,116 single-cell transcriptomes from 35 primary STAD samples alongside independent bulk cohorts, our study transitioned from descriptive profiling to identifying causal regulatory networks. We delineated meta-program 2 (MP2), a malignant-specific transcriptional state quantitatively coupled to immune evasion, and isolated RNASE1 as its core molecular mediator.

As a prominent member of the pancreatic-type ribonuclease A superfamily, RNASE1 has been historically implicated in modulating endothelial responses, systemic inflammation (32), and acting as an oncogenic driver in other solid malignancies (33). Its role in STAD, particularly in relation to malignant-cell programs and immune-related phenotypes, remains insufficiently defined. Our *in vitro* assays showed that RNASE1 knockdown reduced proliferation, migration, and invasion, supporting its role as a potential contributor to malignant cellular phenotypes.

Beyond these intrinsic malignant shifts, RNASE1 fundamentally rewires the tumor microenvironment to establish an immunosuppressive niche. Transcriptomic deconvolution revealed that elevated RNASE1 expression orchestrates an “immune-excluded” state. This microenvironment is characterized by abundant but functionally impaired CD8+ T cell and dendritic cell infiltration, which is neutralized by widespread upregulation of co-inhibitory checkpoints (e.g., PDCD1, CTLA4) (34). Consequently, RNASE1 exerts a distinct dual functionality: it directly fuels intrinsic malignant cell growth while simultaneously shielding the tumor from extrinsic immune surveillance.

This dual biological role provides a translational rationale for positioning RNASE1 as a precision metric to optimize ICB therapeutic strategies. RNASE1 expression aligns strongly with established transcriptomic response signatures and demonstrates cross-cohort predictive validity in ICB-treated populations, offering a mechanistic alternative to markers with limited accuracy in STAD (e.g., PD-L1, TMB) (35). While our multidimensional data unequivocally link RNASE1 to immune escape, deciphering the exact molecular crosstalk between RNASE1-expressing tumor cells and immune populations necessitates future *in vivo* immune-competent modeling. Ultimately, decoding these RNASE1-driven immune-genetic dynamics provides a robust quantitative framework for prognostic stratification and rational immunotherapeutic intervention in advanced STAD.

## Limitations

This study has several limitations. First, most transcriptomic analyses were retrospective and based on public datasets, which may introduce cohort-specific bias. Second, although integrated analysis and validation across independent cohorts were used, residual technical differences among scRNA-seq, RNA-seq, and microarray platforms cannot be fully excluded. Third, the prognostic analyses do not fully establish independence from all standard clinicopathological factors, because complete covariate information was not uniformly available across cohorts. Fourth, multi-omics, proteomic, and biomarker-comparison analyses were not systematically performed in the present revision. Fifth, the *in vitro* functional experiments were limited to two STAD cell lines and did not include pharmacological RNASE1 blockade. Sixth, immune-related conclusions were mainly inferred from transcriptomic deconvolution, immunomodulator correlation, and ICB-response signatures; *in vivo* immune-competent models are needed to directly test whether RNASE1 regulates anti-tumor immunity. Finally, prospective ICB-treated STAD cohorts are required before RNASE1 can be applied as a clinical predictor of checkpoint blockade response.

## Data Availability

The original contributions presented in the study are included in the article/**Supplementary Material**. Further inquiries can be directed to the corresponding author.

## Supplementary material

Supplementary Figure 1 Pan-cancer univariate Cox analysis of RNASE1 across 33 TCGA cancer types.
