# Supplementary figures and images for: Tumor-intrinsic immune-genetic dynamics identify RNASE1 as an immune-evasion-associated biomarker and predictor of checkpoint blockade response in gastric adenocarcinoma
Source: Front Immunol. 2026 Jun 30;17:1884482. doi: 10.3389/fimmu.2026.1884482 (PMC13364911; doi:10.3389/fimmu.2026.1884482)

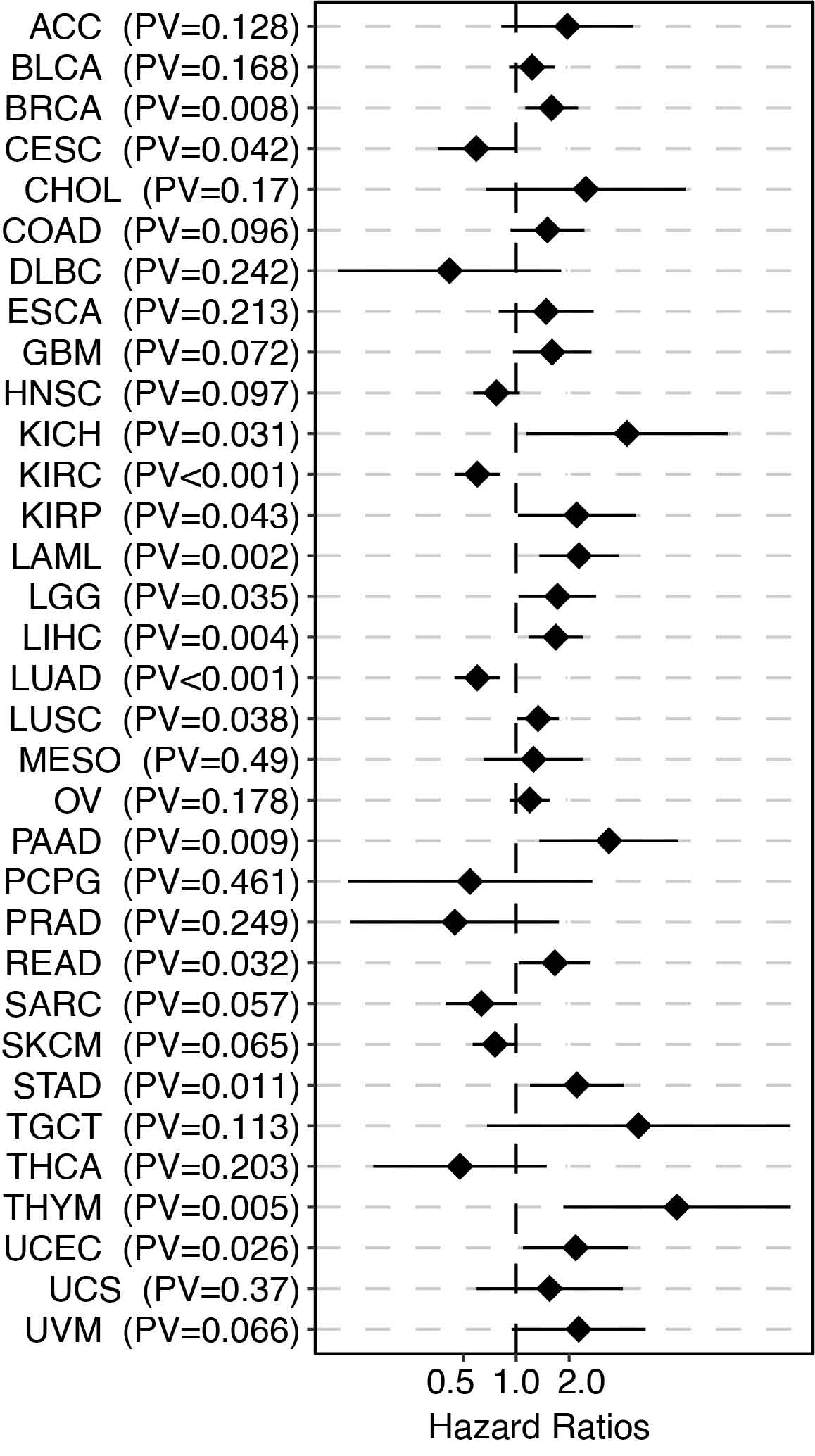


Figure S1. Pan-cancer univariate Cox analysis of RNASE1 across 33 TCGA cancer types.

Supplement: Supplementary Figure 1 — Pan-cancer univariate Cox analysis of RNASE1 across 33 TCGA cancer types. [file DataSheet1.docx]
